# Clinical utility of fetal echocardiography: an Egyptian center experience

**DOI:** 10.1186/s43044-021-00196-z

**Published:** 2021-08-19

**Authors:** Marwa Moustapha Al-Fahham, Nada Ayman Gad, Ahmed Ramy Mohamed Ramy, Nevin Mamdouh Habeeb

**Affiliations:** 1 Pediatric Department, Pediatric Cardiology Unit, Faculty of Medicine, Ain Shams University, Ramsis Street, Abbasia, Cairo, 11566 Egypt; 2 Ultrasound Special Care Unit for the Fetus, Faculty of Medicine, Ain Shams University, Ramsis Street, Abbasia, Cairo, 11566 Egypt; 3 Present Address: Al-Salam International Hospital, Bneid Al Gar, Kuwait City, Kuwait

**Keywords:** Fetal echocardiography, Congenital heart disease, Fetal arrhythmia, Prenatal diagnosis, Perinatal risk factors

## Abstract

**Background:**

The impact of early diagnosis of fetal cardiac abnormalities on the postnatal outcome has been controversial in literature. We aimed to evaluate the role of fetal echocardiography (FE) as a diagnostic tool for early detection and proper management of fetal cardiac abnormalities, study the indications of referral and detect the perinatal outcome in our institution.

**Results:**

This is a cross-sectional observational and descriptive study that included one hundred and one singleton pregnant women (101 fetuses) who were referred for FE over a period of one year. Indications for referral and perinatal risk factors were documented. FE and postnatal transthoracic echocardiography were done. Fetal cardiac abnormalities were detected in 46.5% of cases. Congenital heart defects (CHDs) in 34.6%, fetal arrythmias in 9.9%, cardiomyopathy in 2.9% and cardiac mass (Rhabdomyoma) in 1% (combined structural and rhythm abnormalities were observed in two fetuses). Of the CHDs, complex heart lesions were diagnosed in 57.1%, common atrioventricular canal in 28.6% and conotrunchal anomalies in 14.3%. Of the ten cases with fetal arrythmias, five fetuses had tachyarrhythmias, four had ectopics and one fetus had congenital heart block in association with maternal lupus. The indications for referral were abnormal obstetric ultrasound (52.5%), maternal medical illnesses (23.8%), multiple neonatal deaths (13.9%) and positive family history of CHD (10.9%). The number of fetuses with cardiac abnormalities was significantly higher than those without cardiac abnormalities in mothers not exposed to perinatal risk factors (*p* = 0.009) and was statistically lower in mothers exposed to perinatal risk factors (*p* = 0.005). FE showed 100% accuracy in diagnosing complex lesions, common atrio-ventricular canals, cono-truncal anomalies, cardiac masses and fetal arrhythmias. It missed two cases of tiny muscular ventricular septal defects and one case of aortic coarctation. Cases of fetal supraventricular tachycardia were successfully treated in-utero.

**Conclusions:**

CHDs exist in fetuses with no underlying perinatal risk factors. FE can accurately diagnose most of the cardiac anomalies though few errors remain challenging (aortic coarctation). It also offers a good chance for successful early life-saving management of some types of fetal arrhythmia.

## Background

CHD is the most common congenital anomaly that occurs in 6–12 per 1000 live births [1]. Several risk factors have been incriminated in the pathogenesis of CHD [2]. These include consanguinity [3], maternal medical illnesses [4], teratogenic exposures [5], fetal chromosomal and extra-cardiac abnormalities [6]. However; a major number of cases do exist without any detectable prenatal risk factors [7].

The impact of prenatal diagnosis of CHD on the postnatal outcomes has been controversial. Several studies have shown that it decreased neonatal morbidity and mortality; usually of the duct-dependent types [8] as it provides the opportunity for a controlled elective delivery at a tertiary care center with a specialized perinatal cardiac and cardio-thoracic surgical services [9].Moreover, it helps the detection of concomitant fetal chromosomal and extra-cardiac anomalies, assists in antenatal and postnatal management planning and offers better parental counseling [10]. Other studies found no benefit on neonatal outcomes [11].

FE is also the most widely used diagnostic modality for the detection of fetal arrhythmias [12] and evaluation of its consequences such as valve regurgitation, myocardial dysfunction and development of hydrops fetalis [13].

FE is used to detect cardiac anomalies in high risk specific cases [14]; though its use as a routine screening tool for all pregnancies is still not well-established [15]. Most of the referrals are still based on the presence of abnormal 4 chamber view on obstetric ultrasound scan or the presence of a favoring prenatal risk factor despite that only 10% of the affected children have such an identifiable predisposing factor [16]. In this study, we aimed to evaluate the role of FE as an evolving diagnostic tool in our institution for early detection and proper management of fetal cardiac abnormalities; including fetal arrhythmia and to detect the perinatal outcome of the affected fetuses. We also studied the distribution of the well- known perinatal risks in our community. Up to the best of our knowledge, this is the first Egyptian study that documents the utility of FE in the management of fetuses with suspected cardiac abnormalities.

## Methods

This is a cross-sectional analytical and descriptive study that included 101 singleton pregnant women (101 fetuses) referred for FE over a period of 1 year from November 2014 till October 2015. The study was conducted at the ultrasound special care unit for the fetus at the obstetrics and gynecology hospital in collaboration with the pediatric cardiology and echocardiography unit at a university hospital. Detailed history was taken from all pregnant mothers which included maternal and paternal ages, consanguinity, obstetric history (gestational age (GA), parity, abortions, still births, assisted reproductive technology (ART), neonatal deaths) and family history of CHD, chromosomal or other congenital anomalies. Prenatal risk factors were extracted (active and passive smoking, maternal medical illnesses, infections, medications and exposure to radiation). Indications for referral were reported and the original indication was chosen in patients who fell in more than one referral category.

### Fetal echocardiography

FE was done for the referred cases to detect any structural or rhythm abnormalities in the fetal heart according to the guidelines and standards of the American Society of Echocardiography for performance of fetal echocardiogram [17] using GE Voluson E6 high-end 4DOB/GYN ultrasound machine and transducer probe with a frequency range of 5–8 MHz. A standard two-dimensional (2D), m-mode, color flow (CF), Doppler echocardiography examinations were done. Views included four chamber, five chamber, long axis (left and right ventricular outflow), 3 -vessel and tracheal, ductal, and aortic arch views.

### Neonatal echocardiography (NE)

NE was done within 48 h of delivery; based on the urgency of each individual case; using Vivid 7 machine (GE N-3190, Horton, Norway) and probe 10SGE ultrasound (SN 66958 PD0/USA). The studied population was classified into two groups according to the presence (group A) or absence (group B) of fetal cardiac abnormalities. Structural CHDs were categorized into common AV canal, conotrunchal anomalies, septal defects, complex lesions and coarctation. When abnormalities were found; prognosis and management were discussed with the parents and the referring obstetrician. Arrangement for controlled elective delivery in our tertiary center under the supervision of expert pediatric cardiologists and neonatologists was planned for all fetuses diagnosed with cardiac abnormalities in-utero. In-utero antiarrythmic therapy was offered in some cases with fetal arrhythmia.

### Statistical analysis

Data were analyzed using Statistical Program for Social Science (SPSS) version 18.0. Quantitative data were expressed as mean ± standard deviation (SD). Qualitative data were expressed as frequency and percentage. Independent sample t-test was used to compare two means and Chi-square test was used to compare proportions between two qualitative parameters**.** *P *value of < 0.05 was considered significant. Sensitivity, specificity, positive and negative predictive values and accuracy of FE were calculated to assess its ability to detect the cardiac abnormality in utero and to detect the same cardiac abnormality in the postnatal period.

## Results

The study involved 101 singleton pregnant women (101 fetuses) whose ages ranged from 17 to 42 years with a mean and SD of (28.09 ± 5.19) years. All the referred cases had undergone routine obstetric ultrasound before referral to detect multiple gestation and to diagnose any extra- cardiac abnormality such as hydrops fetalis, increased first trimester nuchal translucency, abnormal fetal heart beats and abnormal four and five chamber views. The timing of the routine obstetric scan ranged from 16 to 20 weeks with a mean and SD of (18 ± 2.5) weeks. Their gestational age (GA) at referral for FE ranged from 16 to 38 weeks with a mean and SD of (25.2 ± 3.4) weeks and 70.5% of the cases were referred after 22 weeks. Their paternal ages ranged from 24 to 48 years with mean and SD of (32.13 ± 5.39) years. The mean values of maternal, paternal and gestational ages were 28.45 ± 5.37 years, 32.43 ± 5.69 years and 29.21 ± 5.99 weeks respectively in group A and 27.78 ± 5.05 years, 31.87 ± 5.15 years and 27.64 ± 4.95 weeks respectively in group B with no statistically significant differences between both groups (*p* > 0.05).

### Indications for referrals and perinatal risk factors

Table 1 shows the indications of FE.

**Table 1:** Indications for referrals

Indications for FE	Number (*n* = 101)	Percentage (%)
Abnormal Obstetric US	53	52.5
Cardiac configuration	46	45.5
Fetal rhythm	7	6.9
Maternal medical illnesses	23	22.8
Diabetes	11	10.9
Lupus	7	6.9
Hypertension	5	4.9
Multiple neonatal deaths	14	13.9
Positive family history of CHD	11	10.9

US: Ultrasound

Sixty-six pregnant mothers (65.3%) had perinatal risk factors for CHD and 35 mothers (34.6%) had not shown any perinatal risk factors and were referred for FE on basis of suspicious routine obstetric scan. One perinatal risk factor was detected in 31 cases (30.7%), two risk factors in 19 cases (18.8%) and three or more risk factors were detected in 16 cases (15.8%). Consanguinity was found in 32 cases (31.7%), maternal exposure to radiation in 13(12.9%), passive smoking in 12 (11.9%), multiple abortions in 8(7.9%), maternal intake of medications in 5 (4.9%) cases (corticosteroid therapy in 3 mothers, anti-epileptic medication in one mother and non-steroidal anti-inflammatory medication on one mother). Two cases (1.9%) had been conceived via assisted reproductive technology.

Of the 53 cases referred on basis of suspicious obstetric scan, thirty eight cases (71.7%) proved to have positive cardiac abnormalities on fetal scanning. On the other hand, nine fetuses who had positive cardiac findings on FE scanning had not revealed any suspicious findings on obstetric ultrasound and were referred for FE screening for the presence of associated risk factors. Of the total population, discrepancies between the routine obstetric scan and the FE findings were encountered in 21 cases (Table 2).

**Table 2:** Discrepancies between routine obstetric ultrasound scan and FE

Case number	Obstetric US	FE	Post natal assessment
*Cases with suspicious Obstetric US and normal FE*			
(18, 55, 61–69)	Suspicious	Normal	Normal
(21) and (56)	Suspicious	Normal	Not done (Lost follow up)
(43)	Suspicious	Normal	Not done (IUFD)
*Cases with positive findings by FE and unsuspicious obstetric US*			
(39)	Normal	Cono-trunchal anomaly	Not done (Lost follow up)
(45,52)	Normal	Complex CHD	Confirmed
(46)	Normal	Complex CHD	Not done (Stillbirth)
(51)	Normal	Common AV canal	Not done (Lost follow up)
(58)	Normal	Prolonged PR interval	confirmed
(72)	Normal	Atrial tachycardia	Not done (Elective termination)
(75, 82)	Normal	Common AV canal	Confirmed

*CHD* congenital heart defect, *FE* fetal echocardiography, *NE* neonatal echocardiography, *US* ultrasound

The number of fetuses with positive cardiac findings was significantly higher than those without cardiac abnormalities in mothers not exposed to any perinatal risk factors and in mothers referred on basis of a suspicious obstetric US (abnormal cardiac configuration or abnormal fetal rhythm) and was significantly lower than fetuses with normal hearts in mothers exposed to perinatal risk factors. Table 3 shows the comparison between the studied groups as regards to the distribution of the perinatal risk factors and the indications of FE.

**Table 3:** Comparison between both groups as regards to the perinatal risk factors and indications for referral

	Group A (*n* = 47)	Group B (*n* = 54)	Chi square *X*^2^	*P*
Abnormal Cardiac configuration by obstetric US	31	15	14.769	0.000*
Fetal arrhythmia by obstetric US	7	–	8.641	0.003*
No perinatal risk factors	23	12	6.914	0.009*
Perinatal risk factors	24	42	7.919	0.005*
One risk factor	14	17	0.34	0.854
Two risk factors	7	12	0.884	0.347
≥ 3 risk factors	3	13	5.899	0.151

*US* ultrasound

*P* < 0.05 considered significant

### Fetal echocardiography

Difficulties in obtaining adequate echocardiographic views were encountered in only 4 cases; two of them due to the anterior position of the fetal vertebral column obscuring the view, one case due to maternal obesity and one case due to inappropriate timing (late referral).

Fetal cardiac abnormalities were detected in 47 fetuses (46.5%). CHDs in 35 (34.6%), fetal arrhythmia in 10 (9.9%), cardiomyopathy in 3 (2.9%) fetuses and cardiac mass (Rhabdomyoma) in only one fetus (1%) [Two cases had combined pathologies (one fetus had common AV canal and SVT, the other had cardiomyopathy and frequent PVCs)]. Of the structural CHDs, complex heart lesions were found in 20 (57.1%), common atrioventricular canal in 10 (28.6%) and conotrunchal anomalies in 5 fetuses (14.3%).Of the fetal arrythmias, five fetuses had tachyarrhythmias, four had ectopics and one fetus had congenital heart block in association with maternal lupus. Combined structural and rhythm abnormalities were observed in two fetuses (Table 4). Follow up FE examinations were needed in 26 cases (25.7%) due to disease progression (23 cases), obscuring fetal position (2 cases) and maternal distress in one case.

**Table 4:** Management decisions and outcome of fetuses with cardiomyopathy, rhythm abnormalities and cardiac mass

Diagnosis	Decision	Outcome
*Cardiomyopathy*		
Case no (35)	Prevention of termination/regular FUP	Resolved intrauterine
Case no (88) Associated with frequent PVCs	Prevention of termination/regular FUP	IUFD
Case no (91)	Prevention of termination/regular FUP	Lost FUP
*Cardiac mass* (Rhabdomyoma)	Prevention of termination/regular FUP	spontaneous regression
*Arrhythmias*		
Case no (41) Frequent PVCs	Prevention of termination/regular FUP	Resolved intrauterine
Case no (50) Atrial fibrillation	Immediate induction of labor	Mother refused, DAMA signed
Case no (58) Congenital heart block	Admission under obstetric care/regular FUP scanning	Arranged for neonatal pacemaker
Case no (70) Atrial flutter with variable conduction	Regular FUP	Lost FUP
Case no (72) Atrial tachycardia	Antiarrhythmic given without improvement	Elective termination due to severe hydrops
Case no (78) Frequent PACs	Regular FUP	Resolved intrauterine
Case no (79) Frequent PACs Case no (84)	Regular FUP	Resolved intrauterine
SVT + common AV canal	Antiarrhythmic /regular FUP	Postnatal Antiarrhythmic therapy
Case no (88)	Discussed above	Discussed above
Case no (95) SVT	Antiarrhythmic/regular FUP	Post natal antiarrhythmic therapy

*DAMA* discharge against medical advice, *FUP* follow up, *IUFD* intra-uterine fetal death, *PAC* premature atrial contraction, *PVC* premature ventricular contraction, *SVT* supraventricular tachycardia

**Fig. 1:**
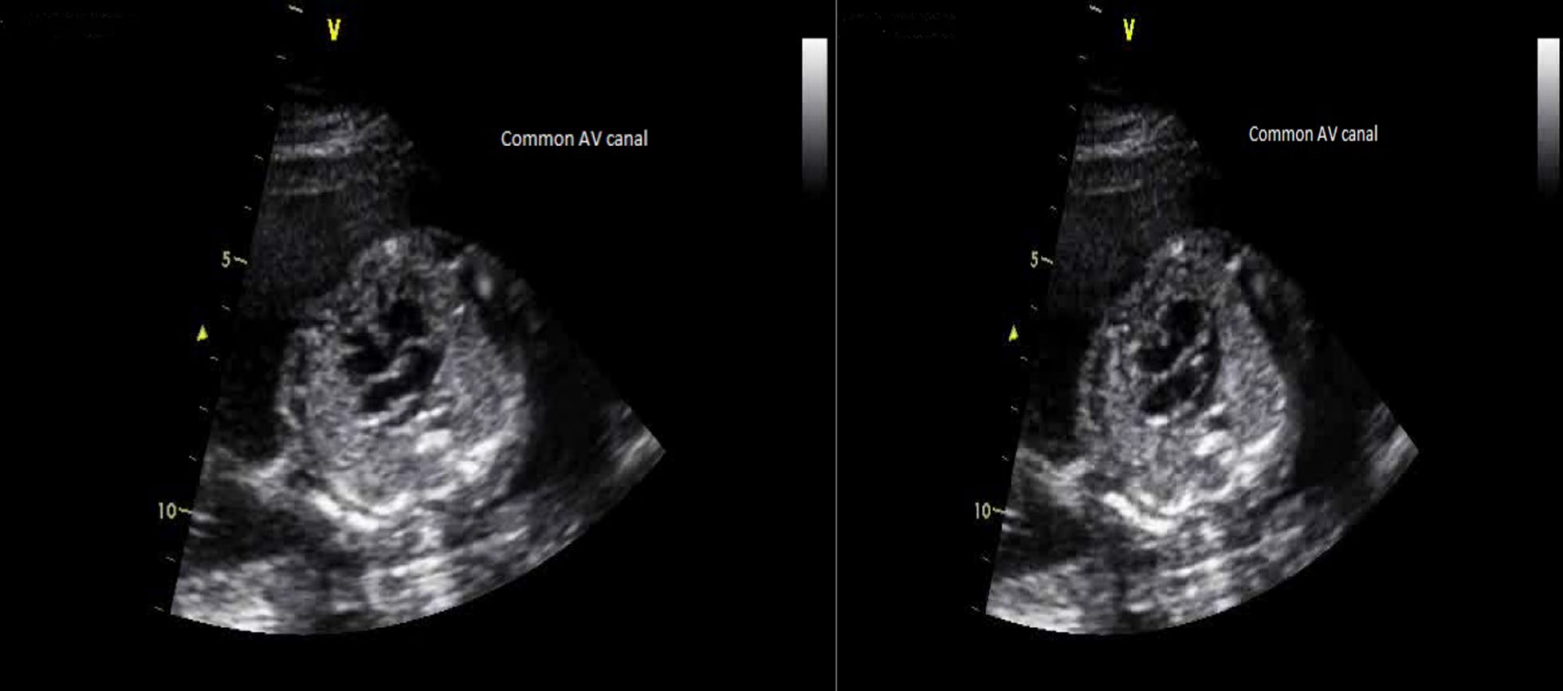
Common AV canal in cases no (75) and (81)

**Fig. 2:**
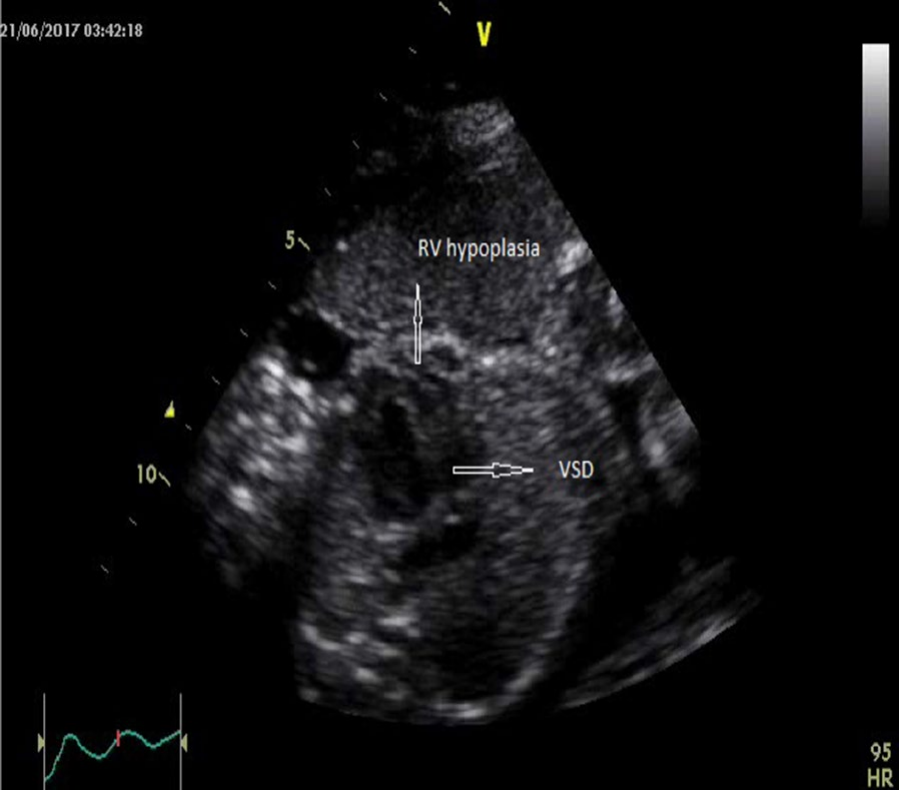
Complex congenital heart disease in case no (98)

**Fig. 3:**
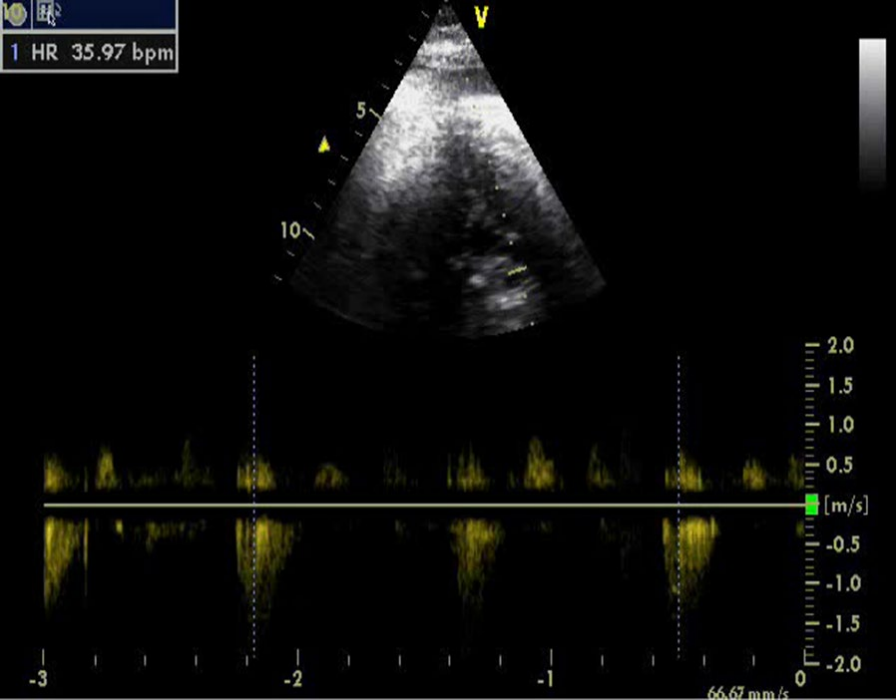
Fetal heart block in an obese lupus mother

Figures 1, 2 and 3 demonstrate some of the encountered fetal cardiac abnormalities.

### Decision and outcome

The decisions taken regarding fetuses with CHD were watchful follow up and arranging for elective delivery in our tertiary care center which offers highly qualified cardiac and cardiothoracic services. Of the total number of population, term delivery occurred in 55 cases, preterm in 2 cases, Intrauterine fetal death (IUFD) in 8 cases, abortion in one case, stillbirth in one case and elective termination in one case. Twenty-seven cases lost follow up, five cases were cured in-utero and discharge against medical advice (DAMA) was signed in one case.

Table 4 shows the decisions taken and outcome of fetuses with arrhythmias, cardiomyopathy and cardiac mass.

Of the twenty fetuses diagnosed with complex heart lesions, three fetuses died in-utero, one was stillbirth, six lost follow up and ten were born alive and received the required postnatal cardiac care. Of the nine fetuses with common AV canal (excluding the case with associated arrhythmia), one died in-utero, three lost follow up and five were born alive. Of those five fetuses with conotrunchal anomalies, two lost follow up and three were born alive.

### Neotnatal echocardiography

NE was done for 57 neonates (21 cases of group A and 36 cases of group B). FE was 100% sensitive and accurate in diagnosing complex heart lesions, atrio-ventricular septal defects, conotrunchal anomalies and cardiac mass.

Table 5 shows the sensitivity, specificity, predictive values and accuracy of FE in the diagnosis of each of the specific fetal cardiac abnormalities (after excluding cases that were cured in-utero, died (IUFD or still birth), electively terminated and cases which lost follow up).

**Table 5:** Comparison between fetal and postnatal echocardiography

	FE	NE	Sensitivity (%)	Specificity (%)	PPV (%)	NPV (%)	Accuracy (%)
Complex CHD	10	10	100	100	100	100	100
Common AV canal*	6	6	100	100	100	100	100
Conotrunchal anomaly	3	3	100	100	100	100	100
Cardiac mass	1	1	100	100	100	100	100

*FE* fetal echocardiography, *PPV* positive predictive value, *NPV* negative predictive value, *NE* neonatal echocardiography

^*^Including the case with associated arrhythmia

Two cases with tiny muscular ventricular septal defects and one case with coarctation of aorta were missed by FE and were diagnosed postnatally. The routine obstetric US scan of the case with aortic coarctation was done at 19 weeks gestation and was unsuspicious of any cardiac abnormalities. The case was referred for FE in the view of maternal history of multiple previous abortions. FE was done at the age of 20 weeks gestation and revealed mild dilatation of the left ventricle. A second look was decided for the case to monitor the disease progression but the mother did not return back for follow up until after delivery when NE revealed mild coarctation.

## Discussion

In our fetal care unit; almost all pregnant women undergo a minimum of one obstetric ultrasound that includes fetal heart scanning based on the four and five chamber views to screen for CHD. A detailed FE scanning by an expert pediatric cardiologist is indicated when findings in these views do not fulfill the normal criteria [18]. Deviation from normality includes abnormal cardiac position or axis, chamber dilatation and or asymmetry, cardiomegaly and fetal arrhythmia [19]. Obtaining only a normal four chamber view is still not sufficient to exclude underlying cardiac anomalies [20], hence; the routine obstetric scan may not be the ideal tool to screen for fetal cardiac abnormalities [21]. FE is more sensitive and more specific in the prenatal detection of CHD when compared to the routine obstetric scanning that can miss a large number of cases [22]. In a study done by Archiron et al. [23], the rate of detection of CHD was raised from 48%; when relying on the four chamber view alone; to 78% when other views were incorporated. Similarly, Carvalho et al. [24] reported a detection rate of 75% with extended screening. In this series, discrepancies between the routine obstetric scan and the FE were encountered in 21 cases. Of which; nine cases showed positive findings by FE though their routine obstetric scan was marked as "Normal". This highlights the need to raise the awareness of the obstetricians and the obstetric ultra- sonographers that limited screening of the fetal heart by the 4- and 5- chamber views may not be sufficient to totally exclude an underlying fetal cardiac abnormality.

In this series, more than half of the cases (52.5%) were referred for suspected cardiac abnormalities on routine obstetric ultrasound (abnormal cardiac configuration in 45.5% and abnormal fetal rhythm in 6.9%) and of the referred cases, the percentage of positive FE scans was 71.7%. In a study done by Meyer-Wittkopf et al. [18], cases referred on basis of suspicious cardiac configuration and abnormal fetal rhythm were (26%) and (5.7%) of their studied population respectively and the percentage of FE scans confirmed positive for CHD was 78%. Also in a study done by Chitra and Vijayalakshmi [25], abnormal obstetric scan was the indication of referral in 26.8% of their studied population. This highlights the role of obstetric US scanning as an important screening tool for fetal structural and rhythm abnormalities, though; unfortunately we were not able to compare between the accuracy of the obstetric and the FE scanning as most of the referral letters included “suspected cardiac views” without mentioning a definitive provisional diagnosis.

Currently, FE is reserved for high risk pregnancies where higher incidence of CHD is traditionally expected [22], though; previous studies reported that most of the cases of CHDs occur in low risk population with no identifiable prenatal risk factors [7, 16, 26]**.** In this series, the number of fetuses with positive cardiac findings was significantly higher than those without cardiac abnormalities in mothers not exposed to any perinatal risk factors and was significantly lower than fetuses with normal hearts in mothers exposed to perinatal risk factors. This matches with Nayak et al. [22] who advocated that FE should be included as a routine antenatal scanning for all pregnancies irrespective of the perinatal risk factors for congenital cardiac anomalies as they found no statistical difference in the incidence of these anomalies between low risk versus high risk pregnancies; moreover, they even encountered a higher number of cases with CHDs in the low risk group.

The ideal timing for prenatal echocardiography is 18–22 weeks gestation [17]. In this series, the mean value of gestational age on first FE scanning was (25.2 ± 3.4) weeks. This compares favorably with previous studies [25, 27, 28].

In this study, we detected cardiac abnormalities in 46.5% of the referred fetuses. CHDs in 34.6%, fetal arrythmias in 9.9%, cardiomyopathy in 2.9% and cardiac mass (Rhabdomyoma) in 1%. Of the CHDs, complex heart lesions were diagnosed in 57.1%, common atrioventricular canal in 28.6% and conotrunchal anomalies in 14.3%. In their study, Chitra and Vijayalakshmi [25] detected CHD in 18.2%, fetal arrhythmias in 3.6% and rhabdomyomas in 0.6% of their studied population. Among their CHD group, complex lesions were detected in 70% of cases. Also, Meyer–Wittkopf et al. [18] detected CHD in 24.5%. The differences in the detection rate between different studies can be attributed to the significant variations in the incidence of CHD that do exist between different populations belonging to different ethnicities [29].

In a study done by Zhang et al. [30], the sensitivity and specificity of FE in detecting CHD was 68.5% and 99.8% respectively whereas, Soongswang et al. [31] detected sensitivity, specificity, positive predictive value, negative predictive value and accuracy of 96.9%, 90.6%, 84.2%, 98.3% and 92.8% respectively. In this series, postnatal studies revealed that FE was able to diagnose correctly all cases of CHD (conotrunchal anomalies, atrioventricular canal, and complex heart lesions), cardiomyopathy, and cardiac mass after exclusion of cases which died, cured in- utero and cases which lost follow up. We encountered three false negative diagnosises; two small restrictive ventricular septal defects (VSDs) and one coarctation of aorta. Neither of our false negative cases had experienced any deteriorating hemodynamic consequences in the postnatal period and they had come only to medical attention in the view of recruitment for neonatal echocardiography confirmation. The types of cardiac lesions missed in our study match favorably with the study done by Meyer-Wittkopf et al. [18] who detected a sensitivity of 98% in the prenatal diagnosis of CHD by FE. The difficulty in the prenatal diagnosis of aortic coarctaion is well-known in literature [32] and had been reported in previous studies [33, 34]. This confirms the need for sequential follow up studies as some cardiac lesions have an evolving nature [35].

Fetal arrhythmias account for nearly 10–20% of total referrals for FE [12]. Most of which are in the form of frequent ectopic beats with the atrial ectopics being much more common than those of ventricular origin. Tachyarrythmias are diagnosed when fetal heart rate is above 180 beats per minute. They include sinus, atrial, supraventricular and ventricular tachycardia [36]. Fetal bradyarrythmia are diagnosed when fetal heart rate is persistently below 100 beats per minute which can be due to blocked atrial bigemeny or atrio-ventricular block or sinus bradycardia (rare) [12]. In this series, we detected arrhythmias in 9.9% of the studied population. Only one case had an underlying CHD. Of the 10 cases with fetal arrhythmias, five cases had tachyarrhythmia, 4 cases had ectopics and one case had congenital heart block in association with maternal lupus. Cases with ectopics had resolved in the intrauterine period except for the one with associated cardiomyopathy which died in-utero. The case with congenital heart block was followed up meticulously; regular follow up scanning showed improvement in the heart rate (> 55/min) and future planning for neonatal pacemaker was arranged prenatally. Both cases of supraventricular tachycardias (SVT) were successfully treated during the fetal life. Successful treatment of fetal arrhythmia in utero and spontaneous resolution of premature atrial contractions had been also reported by Soongswang et al. [31]. Of their 17 cases diagnosed with fetal arrhythmia, Chitra and Vijayalakshmi [25] reported bradycardia in 10 cases, tachyarrythmias in 5 cases, ectopics in two cases and complete heart block in one case with maternal lupus. They also observed lower association between fetal arrhythmia and underlying structural heart diseases.

Early prenatal diagnoses provides the neonate with a better care in-utero and in the post natal period. Moreover; it allows for early family counseling which allows the parents to be psychologically and financially prepared to accept such a child [37] as it offers them time to be fully aware of the pathophysiology of the detected anomaly; also the treating physician will have enough time to explain the severity and discuss the prognosis with parents so they can be able to take a decision regarding the course of pregnancy. Missing such cases on routine obstetric scanning or discovering them at late pregnancy would have rendered decision taking more difficult [22]. Management of a neonate with an antenatal diagnosis of CHD necessitates coordinate collaboration between obstetricians, neonatologists, pediatric cardiologists, fetal echocardiographers and cardio- thoracic surgeons [38, 39].The management plan is tailored for each case putting into consideration the anticipated risk of hemodynamic instability, the available medical resources, presence of feto-maternal complications, the availability and the transportation distance to a specialized cardiac center [39]. Based on our FE findings; delivery in a tertiary care center with availability of pediatric cardiologist for early neonatal echocardiographic confirmation and subsequent management was decided for all cases with cardiac abnormalities. Arrangement for future pacemaker was done for congenital heart block. Successful antiarrhythmic fetal therapy for SVT cases was done. Prevention of termination with strict follow up was the decision taken for cases with expected spontaneous resolution such as ectopics, cardiomyopathy, rhabdomyoma and even the complex structural heart diseases as termination could not have been offered for such cases based on our cultural and religious backgrounds.

Some studies have shown that neonates with CHD diagnosed antenatally tend to be born earlier than expected when compared to those diagnosed postnatally [40]. Though**,** the decision for the delivery timing of a neonate with a prenatal diagnosis of CHD is affected greatly by the presence or absence of maternal or fetal complications, the advantages of term delivery should be always kept in mind [39]. In our study, most of our cases were term deliveries. This reflects the high standard of obstetric care offered to our population.

## Study strengths and limitations

Former studies showed that the outcome of neonates who had been diagnosed prenatally with a serious CHD and had consequently been offered an appropriate management in their early neonatal period had shown a better outcome in comparison with those diagnosed in their postnatal period [41]. Unfortunately, based on our study design which lacked a control group, we were not able to compare between pregnant mothers who had undergone prenatal FE and those who had not as regards to the perinatal outcome; hence we reported the outcome of our cases in a descriptive manner rather than a numerical one.

This study summarizes the current practice in our institution. Unlike many of the previous studies; it did not only include structural cardiac abnormalities but it also included fetal rhythm abnormalities. Moreover it highlighted the perinatal risk factors frequently encountered in our community but unfortunately; it was limited by the number of cases who did not come for follow up, so postnatal confirmation of the underlying anomaly was not done for all the studied cases.

## Conclusions

CHDs exist in fetuses with no underlying perinatal risk factors. FE can accurately diagnose most of the cardiac anomalies though few errors remain challenging regarding the prenatal diagnosis of aortic coarctation. It also offers a good chance for successful early lifesaving management of some types of fetal arrhythmia. It is important to raise the awareness of the obstetricians and the obstetric-sonographers to refer pregnant mothers for FE in an adequate time when indicated. It is also important to improve the training of the obstetric sonographers to adequately screen for CHD with every follow up visit; owing to the evolving nature of some cardiac lesions; and to refer for FE once suspected. Moreover, there is a growing need to increase the clinical skills of the pediatric cardiologists in the field of FE.

## Data Availability

All data generated or analyzed during this study are included in this published article.

## Abbreviations

ART: assisted reproductive technology
CHDs: congenital heart defects
FE: fetal echocardiography
GA: gestational age
IUFD: intrauterine fetal death
SVT: supraventricular tachycardia
US: ultrasound
